# Near-complete genomes from six human coronavirus HKU1-positive samples recovered by metagenomics in coastal Kenya, 2024–2025

**DOI:** 10.1128/mra.00642-26

**Published:** 2026-08-06

**Authors:** Arnold W. Lambisia, Omar K. Nyawa, Grace Maina, Esther N. Katama, Martin Mutunga, Charles N. Agoti

**Affiliations:** 1 Epidemiology and Demography Department, Kenya Medical Research Institute (KEMRI)—Wellcome Trust Research Programme (KWTRP), Kilifi, Kenya; 2 Pwani University 270495, Kilifi, Kenya; DOE Joint Genome Institute, Berkeley, California, USA

**Keywords:** coronaviruses, HKU1, metagenomics, genomes, coastal Kenya

## Abstract

Human coronavirus HKU1 is globally endemic but genomically understudied. We present six near-complete HKU1 genomes from samples collected in coastal Kenya (2024–2025) that fell into genotypes A (*n* = 3) and B (*n* = 3). The data expand the global HKU1 genomic database and will support molecular assay development and phylogeography studies.

## ANNOUNCEMENT

Of the four globally endemic human coronaviruses that circulated prior to the emergence of SARS-CoV-2 in 2019, HKU1 is the least prevalent, causing respiratory illness in both children and adults (1). A member of the family *Coronaviridae*, genus *Betacoronavirus*, the HKU1 genome comprises a positive-sense, single-stranded RNA molecule ~30 kb in length (2). Its sequences fall into three genotypes (A, B, and C) and can recombine (3, 4). Globally representative genomic data are essential for developing new molecular assays and mapping local and global transmission pathways (3, 5). However, as of March 2026, only 380 well-covered (>70%) HKU1 genomes were publicly available, with only a single genome sampled in Africa.

Here, we report six near-complete HKU1 genomes from nasopharyngeal/oropharyngeal swabs collected between August 2024 and September 2025. Samples were collected through our respiratory virus surveillance platforms in coastal Kenya (6). Patients included individuals both with (*n* = 2) and without (*n* = 4) clinical symptoms. Detection of HKU1 followed viral RNA extraction with RNeasy Kit (QIAGEN) and qPCR using QIAGEN Multiplex RT-PCR + R Kit, with a cycle threshold (Ct) value of <35.0 considered positive using previously described primers, probe, and thermocycling conditions (7).

For sequencing, viral RNA was re-extracted using QIAamp Viral Mini-RNA Kit (QIAGEN). Host cytoplasmic and ribosomal RNA were depleted using NEBNext rRNA Depletion Kit (New England BioLabs), followed by TURBO DNAse treatment (Invitrogen). First-strand cDNA was synthesized using SuperScript IV RT with Sol-PrimerA (8), and second strand was synthesized using Klenow fragment (3′-> 5′ exo-) (New England BioLabs) as per the manufacturer’s protocol. PCR amplification was performed using Q5 Hot-Start High-Fidelity 2X Master Mix (New England BioLabs) with Sol-PrimerB (8). Libraries were prepared using the Native Barcoding v14 Kit and sequenced on a GridION platform utilizing an R10.4.1 flow cell. The N50 for the run was 318 bp.

Basecalling was performed using Dorado v7.11.2 with the high-accuracy 400 bp model. Reads were pre-processed using Porechop_abi v0.50 (9) and taxonomically classified using kraken v2.1.3 against the maxikraken2_1903_140GB database using default parameters (10). Betacoronavirus FASTQ reads were extracted using seqtk v1.4-r122 and *de novo* assembled using SPAdes v4.2.0 (parameter; -s) (11). Assembled contigs were then queried against a custom local BLAST database with representative HKU1 reference genomes for each genotype (NC_006577.2, AY884001.1, and DQ415897.1) using BLAST+ v2.16.0 (12). Reference-guided assembly of the extracted FASTQ reads was conducted using minimap2 v2.30-r1287 (13) and Medaka v2.1.1 with positions of zero depth masked with default parameters. Multiple sequence alignment was performed using MAFFT v7.526 (14), and phylogenetic trees were generated using IQ-TREE 3 v3.1.1 (parameters; -T AUTO, -B 1000, and -m TEST) (15).

**Fig 1:**
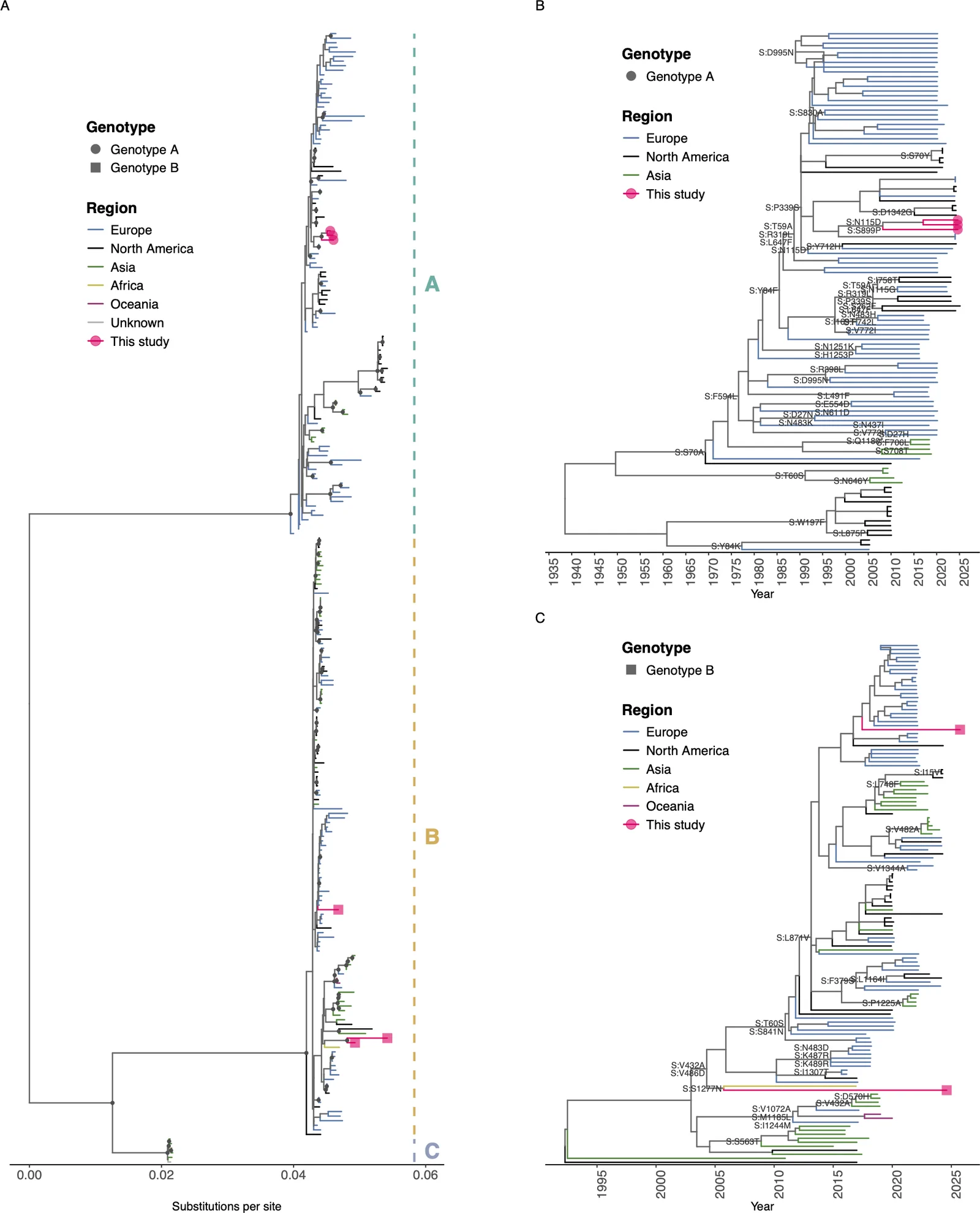
Global phylogenetic context of the recovered HKU1 genomes. (**A**) A maximum likelihood phylogenetic tree showing the genetic relatedness of HKU1 genomes from coastal Kenya (*n* = 6) to global sequences (*n* = 247). The phylogeny branches are colored by geographic region. Global HKU1 genomes included only those with a genome coverage of >70%. The coastal Kenya sequences are colored tip points and shaped by genotype. Filled node circles denote bootstrap support of 100. Panels** B and C** show time-resolved phylogenetic trees for HKU1 genotypes A and B estimated with TreeTime (16) under a strict molecular clock (5). Non-synonymous changes in the spike protein are annotated to the ancestral nodes carrying at least one substitution. One genotype B sequence with 80.5% genome coverage presented as an outlier and was removed from the time-resolved tree analysis.

Genome coverage for the recovered sequences ranged from 80.5% to 99.5% (Table 1). The sequences fell into genotype A (*n* = 3) and B (*n* = 3) (Fig. 1). Pairwise nucleotide differences ranged from 21 to 35 for genotype A and 126 to 219 for genotype B. Non-synonymous mutations in the Spike protein were observed in both genotype A (reference: NC_006577.2, *n* = 6; S:S412F, S:C498Y, S:D526Y, S:S814Y, S:S818Y, and S:S906P) and B (reference: AY884001.1, *n* = 10; S:R396P, S:R397I, S:V486E, S:D507G, S:T509F, S:W515L, S:T527P, S:A747T, S:A747N, and S:K809V). Notably, one sequence contained a single mutation (T > C) within the forward diagnostic primer region.

**TABLE 1:** Details of the samples and HCoV-HKU1 genomes recovered from six respiratory samples collected in coastal Kenya (2024–2025)^*a*^

Strain	Study	Genotype	Collection date	qPCR Ct value	Age (years)	Sex	Clinical status	Total reads	HKU1 reads	Genome length	GC content	Average depth	Genome coverage	GenBank accession	SRA accession
HKU1_KLF_GA_10000	ResViRe	A	15/08/2024	24.145	4.0	Male	Asymptomatic	455,463	16,134	29,309^*d*^	31.9	53	97.9	PZ311792	SRR38128216
HKU1_KLF_GA_10001	ResViRe	A	19/08/2024	26.501	30.3	Female	Asymptomatic	468,844	15,312	25,511^*e*^	31.9	50	85.2	PZ311793	SRR38128215
HKU1_KLF_GA_10002	ResViRe	A	19/08/2024	23.86	1.6	Female	Asymptomatic	809,351	4,853	29,732^*d*^	32	17	99.4	PZ311794	SRR38128214
HKU1_KLF_GB_10003	HF	B	12/08/2024	24.75	34.0	Female	Symptomatic^*b*^	450,856	8,832	29,653^*f*^	32	31	99.5	PZ311795	SRR38128213
HKU1_KLF_GB_10004	HF	B	12/08/2024	28.134	7.0	Female	Symptomatic^*c*^	454,979	736	23,991^*g*^	32.2	3	80.5	PZ311796	SRR38128212
HKU1_KLF_GB_10005	ResViRe	B	29/09/2025	27.223	4.1	Male	Asymptomatic	523,089	4,270	27,854^*d*^	32.1	18	93.5	PZ311791	SRR38128211

^*a*^ qPCR Ct, real-time polymerase chain reaction cycle threshold; HF, health facility outpatient acute respiratory illness surveillance; ResViRe, respiratory virus reinfections community study that collects samples regardless of symptom status.

^*b*^ Fever, cough, nasal discharge, and sore throat.

^*c*^ Fever and cough.

^*d*^ All genes are complete coding sequences except *ORF1ab*.

^*e*^ All genes are complete coding sequences except *ORF1ab*, *S*, and *ORF4*.

^*f*^ All genes are complete coding sequences except *ORF1ab* and *S*.

^*g*^ All genes are complete partial coding sequences except *E*, *N*, and *N2*.

## Data Availability

The FASTQ files were deposited in the Sequence Reads Archive database (SRR38128211–SRR38128216) and final consensus genomes deposited in the GenBank database (PZ311791–PZ311796).
